# Association between hedonic hunger and body-mass index versus obesity status

**DOI:** 10.1038/s41598-018-23988-x

**Published:** 2018-04-11

**Authors:** Gabriela Ribeiro, Marta Camacho, Osvaldo Santos, Cristina Pontes, Sandra Torres, Albino J. Oliveira-Maia

**Affiliations:** 1 Champalimaud Clinical Centre, Champalimaud Centre for the Unknown, Av. de Brasília, Doca de Pedrouços, 1400-038 Lisboa, Portugal; 2 Lisbon Academic Medical Centre PhD Program, Faculdade de Medicina, Universidade de Lisboa, Avenida Professor Egas Moniz, 1649-028 Lisboa, Portugal; 3 Instituto de Saúde Ambiental, Faculdade de Medicina, Universidade de Lisboa, Avenida Professor Egas Moniz, 1649-028 Lisboa, Portugal; 4 Instituto de Medicina Preventiva e Saúde Pública, Faculdade de Medicina, Universidade de Lisboa, Avenida Professor Egas Moniz, 1649-028 Lisboa, Portugal; 5 Psychiatry and Mental Health Clinic, Centro Hospitalar de São João, Alameda Prof. Hernâni Monteiro, 4200-319 Porto, Portugal; 6 Faculdade de Psicologia e de Ciências da Educação, Universidade do Porto, Rua Alfredo Allen, 4200-135 Porto, Portugal; 7 Centro de Psicologia da Universidade do Porto, Rua Alfredo Allen, 4200-135 Porto, Portugal; 8 Department of Psychiatry and Mental Health, Centro Hospitalar de Lisboa Ocidental, Rua da Junqueira, 126, 1340-019 Lisboa, Portugal; 9 NOVA Medical School | Faculdade de Ciências Médicas, Universidade Nova de Lisboa, Campo Mártires da Pátria 130, 1169-056 Lisboa, Portugal; 10 Champalimaud Research, Champalimaud Centre for the Unknown, Av. de Brasília, Doca de Pedrouços, 1400-038 Lisboa, Portugal; 11 Present Address: John van Geest Centre for Brain Repair, Department of Clinical Neurosciences, School of Clinical Medicine, University of Cambridge, Cambridge, CB2, 0SP, UK

## Abstract

Obesity-associated differences in hedonic hunger, while consistently reported, have not been adequately quantified, with most studies failing to demonstrate strong correlations between Body Mass Index (BMI) and hedonic hunger indicators. Here, we quantified and assessed the nature of the relationship between hedonic hunger and BMI, in a cross-sectional study using the Portuguese version of the PFS (P-PFS) to measure hedonic hunger. Data were collected from 1266 participants belonging to non-clinical, clinical (candidates for weight-loss surgery) and population samples. Across samples, significant but weak positive associations were found between P-PFS scores and BMI, in adjusted linear regression models. However, in logistic regression models of data from the clinical and non-clinical samples, the P-PFS Food Available domain score was significantly and robustly associated with belonging to the clinical sample (OR = 1.8, 95%CI: 1.2–2.8; p = 0.008), while in the population sample it was associated to being obese (OR = 2.1, 95%CI: 1.6–2.7; p < 0.001). Thus, hedonic hunger levels are associated with obesity status with the odds of being obese approximately doubling for each unit increase in the P-PFS Food Available score.

## Introduction

Obesity is thought to result from gene-environment interactions, mediated by complex neuronal and hormonal systems^1,2^. Overeating plays a fundamental role in obesity, with significant inter-individual variability in sensitivity to food cues^3–6^, that may condition differential predispositions for obesity^7,8^. Importantly, overeating can be triggered by frequent consumption of palatable and energy-dense foods, even in the absence of an energy deficit^9^, and has been proposed to result, at least in part, from the reward associated with food consumption^10^. In fact, the consumption of such palatable and energy-dense foods, ubiquitous in the modern food environment^11^, activates dopaminergic reward circuits in the brain, through both oral and postingestive mechanisms^12,13^.

Consumption of highly palatable foods has also been shown, in itself, to induce plastic modifications of dopaminergic circuits, and induce compulsive food consumption in rodents^14^. This is thought to explain, at least in part, lower striatal dopamine D2 receptor (DA D2R) availability in severely obese patients, when compared with non-obese subjects^15,16^. There is also evidence supporting a shift in food reward, from the ingestion of palatable foods to sensory cues that precede consumption, with the development of obesity^17^. Specifically, obese compared with normal-weight individuals have shown greater responsivity in reward circuitry to food cues^18,19^, but less activation of this system during palatable food intake^20,21^.

The sensitivity to the rewarding properties of highly palatable foods in the environment, described as hedonic hunger^22^, can be assessed by the Power of Food Scale (PFS)^23,24^. Such hedonically driven motivation to eat has been extensively associated to obesity^24^, with higher hedonic hunger in severely obese patients relative to non-obese controls^25,26^, as well as to obesity-related behavioral patterns, such as selective attention to food cues^27^, food cravings^28^, binge eating disorder (BED)^10^ and self-reported overeating^29^. Furthermore, for both normal weight volunteers^30^ and obese subjects^31^, it has been shown that individuals with increased PFS scores have higher susceptibility to experience loss of control (LOC) over eating, a defining feature of binge eating and predictor of future weight gain^30^. For instance, PFS scores prospectively predicted the development of LOC eating episodes at 1-year follow-up in a sample of college students^30^, highlighting a potential role of hedonic hunger not only as a characteristic of obese individuals, but also in the risk of developing obesity. Finally, prospective studies of obese patients undergoing Roux-en-Y gastric bypass surgery have shown significant reductions in PFS scores after this procedure^32,33^.

Despite the evidence supporting a relationship between hedonic hunger and the etiology of weight gain and/or of excess weight maintenance, most studies do not report significant correlations between BMI and PFS scores^23,25,34–38^. Thus, while hedonically driven motivation to eat is clearly associated with obesity, this may reflect a non-linear relationship with BMI. Similarly, non-linear associations with BMI have been described for other reward-related measures, spanning from food addiction^39^ and BED^39^ to measures of dopaminergic function^40^. Such non-linearity has been proposed to result from the existence of discrete differences across obesity categories^39,41^.

However the nature of the relationship between hedonic hunger and body mass index (BMI) remains unclear and has not been adequately quantified. In this study we aimed to assess the nature of the relationship between these two measures using a non-clinical sample and a multicenter clinical sample of obese patients. Associations between PFS scores and BMI, as well as obesity status defined by World Health Organization BMI cut-offs, were thus tested, and then confirmed in a community-based sample, representative of the general adult population in Portugal. Considering the framework described above for other reward-related measures, we hypothesized that the association between hedonic hunger and BMI follows a non-linear trend.

## Methods

### Study design and samples

Data for the present study were derived from three groups of adult subjects: candidates for weight-loss surgery (clinical sample), a group of college and vocational school students (non-clinical sample), and a representative sample of the population in Portugal (population sample). Participants were 18 years or older. The clinical sample included obese participants (BMI ≥ 30 kg/m^2^), and was recruited consecutively at obesity surgery clinics, in two Portuguese hospitals (Hospital São João and Hospital Espírito Santo de Évora). The non-clinical sample was recruited from three education institutions using non-probabilistic sampling. The population sample was obtained through a randomized population mail-based survey organized by the main Portuguese consumer association (DECO PROTESTE). Full details regarding recruitment of the study sample are reported elsewhere^42^. The Ethics Committees at the Champalimaud Foundation, University of Lisbon School of Medicine, University of Évora, and Hospital S. João (Porto) approved the study protocol. Written informed consent was obtained from all subjects and the study was conducted in accordance with the World Medical Association declaration of Helsinki^43^.

### Measures

Hedonic hunger was assessed using the PFS, a self-report questionnaire consisting of 15 items that assess individual differences in the psychological impact of an environment with high availability of highly palatable foods. The PFS total score is calculated by the mean of the 15 items. The scale includes 3 dimensions of proximity to food derived from factor analyses conducted both with normal weight^23^ and overweight/obese samples^24^: (1) Food Available in the environment but not physically present, (2) Food Present but not yet tasted and (3) Food Tasted but not yet consumed. The score for each dimension is calculated by the average of its items. We used a validated Portuguese version of the PFS (P-PFS), with the same number of items as the original PFS and excellent psychometric properties^42^.

Information regarding demographics (age, gender and education) and anthropometry (height and weight) was collected. In the non-clinical and population samples, data were self-reported in response to standardized questionnaires. For the clinical sample, a clinician interviewed the subjects and a digital scale and stadiometer (Seca, Hamburg, Germany) were used to assess weight and height (measured to the nearest 0.1 kg and 0.1 cm, wearing minimal clothes and no shoes), according to standardized procedures^44^. BMI was calculated as weight (kg) divided by the square of the height (m)^45^ and categorized into normal weight (18.5–24.9 kg/m^2^), overweight (25.0–29.9 kg/m^2^) or obesity (≥30.0 kg/m^2^)^46^. Regarding obesity status, “obese” was defined as having a BMI of 30.0 kg/m^2^ or greater and “non-obese” a BMI below 30.0 kg/m^2^.

### Statistical analysis

Data analysis was performed using SAS software (Version 9.2, SAS Institute, Cary, NC, USA), SPSS (Version 21.0, SPSS Inc., Chicago, IL, USA), and GraphPad Prism (Version 6.0, GraphPad Software, CA, USA). A 5% significance level (*p* < 0.05) was considered. Continuous measurements, presented as mean ± standard error of the mean, were normally distributed according to analysis of kurtosis, skewness and comparison between mean and median. Pearson’s r correlation coefficients were used to assess the unadjusted relationship between BMI and P-PFS scores and independent samples t-test was used to test group differences in P-PFS scores. Effect sizes were determined using Cohen’s d, calculated using the following formulae^47^:

$${\rm{d}}=2{\rm{r}}/\surd (1-{r}^{2})\,{\rm{f}}{\rm{o}}{\rm{r}}\,\text{Pearson}{\textstyle \mbox{'}}s\,{\rm{r}};\,{\rm{d}}={{\rm{M}}{\rm{e}}{\rm{a}}{\rm{n}}}_{1}-{{\rm{M}}{\rm{e}}{\rm{a}}{\rm{n}}}_{2}/{\rm{S}}{\rm{t}}{\rm{a}}{\rm{n}}{\rm{d}}{\rm{a}}{\rm{r}}{\rm{d}}\,{{\rm{D}}{\rm{e}}{\rm{v}}{\rm{i}}{\rm{a}}{\rm{t}}{\rm{i}}{\rm{o}}{\rm{n}}}_{{\rm{p}}{\rm{o}}{\rm{o}}{\rm{l}}{\rm{e}}{\rm{d}}}{\rm{f}}{\rm{o}}{\rm{r}}\,{\rm{t}}-{\rm{t}}{\rm{e}}{\rm{s}}{\rm{t}}$$

Sequential multiple linear regression models were performed to evaluate the relationship between BMI and P-PFS scores, when adjusting for age, gender and education level. Model assumptions were tested by analysis of residuals and influence diagnostics were conducted using Cook’s distance. The associations between group status (clinical vs. non-clinical) or obesity status (obese vs. non-obese) and P-PFS scores were examined using sequential multivariable logistic regression models and expressed by odds ratios (OR) and 95% confidence intervals (CI). Age, gender and education-adjusted prevalence rate ratios (PR) were estimated using multivariable log-binomial regression, in order to assess potential overestimation of the PR by the OR. For all regression models, data transformations and polynomial models were used to test fit for continuous variables.

### Data availability statement

Data regarding the current study is available under reasonable request.

## Results

Demographic information of the clinical, non-clinical and population samples is summarized in Table 1. Women comprised 66.5% of the non-clinical sample and for most participants (82.4%) BMI was under 25 kg/m^2^. In the clinical sample, the gender distribution was even more skewed towards a predominance of women than the other two groups (87.7% women). As expected, BMI was higher in this group. Furthermore, contrary to the non-clinical and the population samples, most of the participants (55.3%) had a low educational level (i.e., 9 or fewer years of formal education). In the population sample, the overall estimated prevalence of obesity was 12.8% (12.8% for women and 12.7% for men).

**Table 1:** Demographic information of the non-clinical, clinical and population samples.

Variable	Non-clinical sample N = 278		Clinical sample N = 123		Population sample N = 865	
	Range	Mean ± SEM	Range	Mean ± SEM	Range	Mean ± SEM
Age (years)	18–50	23.1 ± 0.39	22–71	43.5 ± 0.96	18–74	48.3 ± 0.51
Education (years)	4–17	11.3 ± 0.13	1–20	9.7 ± 0.42	3–24	12.7 ± 0.15
BMI (Kg/m^2^)	16.6–38.6	22.7 ± 0.2	32–61.5	43.4 ± 0.55	15.6–41.1	25.6 ± 0.14
Gender (% male)	33.5%		12.3%		44%	

**Figure 1:**
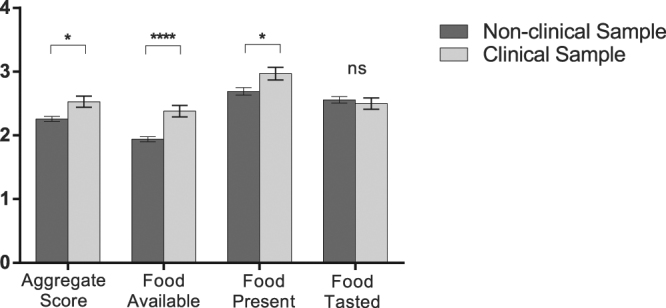
P-PFS Scores for the non-clinical and clinical samples. P-PFS Aggregate and dimension scores (mean ± standard error of the mean) are shown for the clinical (n = 123) and non-clinical (n = 278) samples. Mean scores were compared between the two samples and found to be significantly different in all cases except for the Food Tasted dimension score (t-tests, *p < 0.05, **p < 0.01, ***p < 0.001). P-PFS: Portuguese version of the Power of Food Scale.

As expected, comparison of P-PFS scores between non-clinical and clinical samples revealed significant unadjusted differences between the two groups for the Aggregate Score (t_398_ = −3.1, *p* = 0.002, d = 0.34) as well as for the Food Available (t_398_ = −4.6, *p* < 0.001, d = 0.52) and for Food Present (t_398_ = −2.4, *p* = 0.02, d = 0.27) P-PFS dimension scores, with higher values for the clinical sample. The Food Tasted score, however, did not differ between the clinical and non-clinical samples (t_398_ = 0.6, *p* = 0.5, d = −0.07; Fig. 1). When considering the non-clinical and clinical samples separately, correlations between BMI and the P-PFS Aggregate Score were non-significant (respectively *r* = 0.07, *p* = 0.2, d = 0.14 and *r* = −0.1, *p* = 0.2, d = −0.20), as were correlations with the dimension scores (−0.15 < *r* < 0.11, 0.1 < *p* < 0.9, −0.30 < d < 0.22). However, when the non-clinical and clinical samples were considered jointly (n = 401), significant but weak positive correlations were found between BMI and the P-PFS Aggregate Score (*r* = 0.14, *p* = 0.007, d = 0.28) as well as for the Food Available dimension score (*r* = 0.21, *p* < 0.0001, d = 0.43). Given that non-probabilistic sampling was used for both of these samples, with likely selection bias, we tested if these associations subsisted when adjusting for age, gender and education, using sequential multiple linear regression models (models 1–5, Supplementary Table 1). In these models, the P-PFS Food Available score, but not the Aggregate Score, conserved a significant association with BMI (respectively: β = 1.02, *p* = 0.01; β = 0.56, *p* = 0.2), but it added only marginal predictive power to a baseline model with demographic variables only (R^2^ = 0.57 for both models; see Supplementary Table 1 for details). In addition, when group status (clinical vs. non-clinical) was added to the model showing a significant association between BMI and the P-PFS Food Available score, the association was weaker and no longer significant (β = 0.13, *p* = 0.6; data not shown). These results suggest that the linear relationship between P-PFS scores and BMI, found when considering the clinical and non-clinical samples collectively, is moderated mostly by group differences in P-PFS Food Available scores.

**Figure 2:**
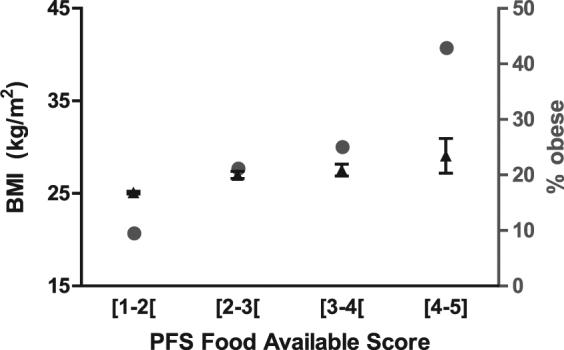
BMI and obesity prevalence in the population sample, according to P-PFS Food Available score categories. Participants in the population sample were divided into 4 groups according to the P-PFS Food Available score ([1−2[, n = 634; [2−3[, n = 123; [3−4[, n = 52; [4−5], n = 14) to assess the distribution of BMI (mean ± standard error of the mean, left y-axis, black symbols) and the proportion of obese individuals (right y-axis, grey symbols). While the mean BMI increased according to the P-PFS Food Available score, this increase was moderate, with BMI ranging from 25.1 ± 0.2 kg/m^2^ in the group with the lowest scores, to 29.1 ± 1.9 kg/m^2^ in the group with the highest scores. However, the prevalence of obesity in each group increased quite dramatically across groups, from 9.5% in the group with the lowest scores, to 42.9% in the group with the highest scores.

To confirm these findings, analyses were repeated with data collected in a larger sample, representative of the general population. In accordance with prior literature and the findings reported above, P-PFS Aggregate scores differed significantly between non-obese (BMI < 30, n = 718) and obese (BMI ≥ 30, n = 105) subgroups (respectively 1.9 ± 0.02 and 2.2 ± 0.09; t_821_ = −4.7, *p* < 0.0001, d = 0.43). Significant obesity-dependent differences were also found for the Food Available (1.6 ± 0.03 and 2.0 ± 0.1; t_821_ = −5.7, *p* < 0.0001, d = 0.56), Food Present (2.2 ± 0.04 and 2.5 ± 0.1; t_821_ = −2.7, *p* = 0.008, d = 0.30) and Food Tasted dimension scores (2.2 ± 0.03 and 2.4 ± 0.09; t_821_ = −2.6, *p* = 0.008, d = 0.24). Regarding associations between P-PFS scores and BMI, we found weak positive correlations for the P-PFS Aggregate (*r* = 0.2, *p* < 0.0001, d = 0.40), Food Available (*r* = 0.23, *p* < 0.0001, d = 0.47), Food Present (*r* = 0.14, *p* < 0.0001, d = 0.28) and Food Tasted (*r* = 0.11, *p* = 0.002, d = 0.22) dimension scores. Importantly, while the significance of correlations between BMI and P-PFS scores were mostly conserved when analyses were repeated in the non-obese and obese subgroups, presumably reflecting enhanced power from large sample sizes, the correlation coefficients were reduced, particularly for the non-obese sample (P-PFS Aggregate: *r* = 0.11, *p* = 0.003, d = 0.22; P-PFS Food Available: *r* = 0.12, *p* = 0.002, d = 0.24; P-PFS Food Present: *r* = 0.11, d = 0.22, *p* = 0.004; P-PFS Food Tasted: *r* = 0.05, *p* = 0.2, d = 0.1). Nevertheless, associations between BMI and P-PFS scores persisted in multiple linear regression models adjusting for gender, age and education (respectively: β = 1.41, β = 1.59, β = 0.67, and β = 0.63, *p* < 0.0005 for all; models 6–10; see Table 2). In these sequential regression models, addition of P-PFS scores to the base model (model 6, adjusted R^2^ = 0.06) increased the ability of the models to predict BMI from 1% (P-PFS Food Tasted dimension, model 10, adjusted R^2^ = 0.07) to 8% (P-PFS Food Available dimension, model 8, adjusted R^2^ = 0.14). In exploratory analyses, when obesity status (obese vs. non-obese) was added to models 7–10, BMI-PFS associations were weaker (β = 0.71, *p* < 10^−4^; β = 0.78, *p* < 10^−4^; β = 0.38, *p* < 10^−4^; β = 0.30, *p* = 0.01; respectively for the Aggregate, Food Available, Food Present and Food Tasted scores) and the contribution of P-PFS scores added to base model, in terms of BMI prediction, was also less expressive (no more than 0.3%; data not shown). These results in the population sample thus confirm that the relationship between P-PFS scores and BMI is moderated mainly by group differences in P-PFS scores rather than a true linear relationship between the two variables (Fig. 2).

**Table 2:** Associations between BMI and P-PFS scores, tested in the population sample using sequential multivariable linear regression models.

Variable	Model 6 (R^2^ = 0.06)		Model 7 (R^2^ = 0.12)		Model 8 (R^2^ = 0.14)		Model 9 (R^2^ = 0.08)		Model 10 (R^2^ = 0.07)	
	β	p	β	p	β	p	β	p	β	p
Gender	−0.07	0.8	−0.19	0.5	−0.17	0.5	−0.13	0.7	−0.15	0.6
Age	0.04	<0.001	0.06	<0.001	0.06	<0.001	0.05	<0.001	0.05	<0.001
Education	−0.14	<0.001	−0.13	<0.001	−0.13	<0.001	−0.13	<0.001	−0.13	<0.001
P-PFS - Aggregate			1.41	<0.001						
P-PFS - Food Available					1.59	<0.001				
P-PFS - Food Present							0.67	<0.001		
P-PFS - Food Tasted									0.63	0.0002

Given the association between obesity and hedonic hunger, we proceeded with quantifications of the association between obesity status and P-PFS scores, when adjusting for demographic variables (age, gender and education level), using multivariable logistic regression models. In the models concerning the clinical and non-clinical samples considered jointly (models 11–15; Supplementary Table 2), only the P-PFS Food Available dimension score was significantly associated to group status (i.e. clinical vs. non-clinical; β = 0.6, *p* = 0.008). The corresponding odds ratio of belonging to the clinical sample was 1.8 for each 1-point score increase (OR = 1.8, 95%CI: 1.2–2.8). These associations were very similar in the population sample (models 16–20; Table 3), in which obese status (obese vs. non-obese) was associated to both Aggregate and Food Available dimension P-PFS scores (0.3 ≤ β ≤ 0.73), with odds ratios of being obese of 1.97 and 2.1 respectively, for each 1-point score increase (OR = 1.97, 95%CI: 1.5–2.6; OR = 2.1, 95% CI: 1.6–2.6). Due to the potential of overestimation of the prevalence ratio when using odds ratios^48^, gender, age and education-adjusted prevalence rate ratios were calculated for P-PFS Aggregate Score (PR = 1.7, 95%CI: 1.4–2.1), Food Available dimension score (PR = 1.7, 95%CI: 1.5–2.1), Food Present dimension score (PR = 1.3, 95% CI: 1.1–1.5) and Food Tasted dimension score (PR = 1.3, 95%CI: 1.1–1.6), and found to very similar to those estimated using logistic regression.

**Table 3:** Associations between obesity status (obese vs. non-obese) and P-PFS scores, tested in the population sample using sequential multivariable logistic regression models.

Variable	Model 16 (c = 0.58)			Model 17 (c = 0.66)			Model 18 (c = 0.68)			Model 19 (c = 0.62)			Model 20 (c = 0.61)		
	β	p	OR (95% CI)	β	p	OR (95% CI)	β	p	OR (95% CI)	β	p	OR (95% CI)	β	p	OR (95% CI)
Gender	0.07	0.75	1.07 (0.7–1.6)	0.002	0.99	1.002 (0.7–1.5)	0.002	0.99	1.002 (0.7–1.5)	0.04	0.8	1.05 (0.7–1.6)	0.02	0.9	1.02 (0.7–1.6)
Age	0.01	0.2	1.01 (0.99–1.03)	0.02	0.03	1.02 (1.002–1.03)	0.02	0.02	1.02 (1.004–1.04)	0.01	0.1	1.01 (0.99–1.03)	0.01	0.1	1.01 (0.99–1.03)
Education	−0.04	0.1	0.96 (0.92–1.01)	−0.03	0.2	0.97 (0.92–1.02)	−0.03	0.2	0.97 (0.92–1.02)	−0.04	0.2	0.97 (0.92–1.01)	−0.04	0.2	0.97 (0.92–1.02)
P-PFS - Aggregate				0.7	<0.01	1.97 (1.5–2.6)									
P-PFS - Food Available							0.73	<0.01	2.1 (1.6–2.6)						
P-PFS - Food Present										0.3	0.004	1.3 (1.1–1.6)			
P-PFS - Food Tasted													0.3	0.007	1.4 (1.1–1.8)

## Discussion

A framework supporting a role for hedonic overeating not only in the maintenance, but also in the development of obesity has been increasingly recognized. Indeed, associations between hedonic hunger and obesity, obesity related eating patterns^24,29,30,36,49–51^ and weight-loss after bariatric surgery^25,32,33^ have led to a conceivable link between hedonic hunger and BMI. However, to date, reports on this association have been inconclusive, with only few studies reporting significant, yet weak, correlations between BMI and PFS scores^24,52^, and most studies reporting no such correlation^23,25,34–38^. The results reported here, while supporting the existence of a weak linear association between hedonic hunger and BMI, strongly suggest that this association results mainly from group-dependent differences in P-PFS scores between obese and non-obese subjects. Furthermore, we confirmed, using two distinct study populations, that the odds of being obese approximately doubles for each point increase of the P-PFS Aggregate or Food Available dimension scores.

In agreement with previous data, we did not find significant P-PFS-BMI correlations in samples of exclusively obese (clinical sample) and mostly non-obese (non-clinical sample) subjects, when these were analyzed separately. Other authors have demonstrated a similar lack of correlation in studies conducted in specific samples, namely healthy students^23^, young adults^34,38^, young adult women^52^, overweight and obese women^49^ and obese patients^24,25^, suggesting this could result from a limited range of BMI values^23,24,34^, or from the fact that PFS might be correlated with overeating^24^ or dieting^38^, rather than BMI per se. However, when we merged the clinical and non-clinical samples, a weak positive BMI-PFS correlation emerged. The group effect suggested by these results was supported by the fact that P-PFS scores differed significantly between the two groups, with higher scores for obese individuals. Furthermore, the effect sizes of the correlational analyses between P-PFS and BMI were similar or lower to those of the categorical analyses comparing non-clinical and clinical samples, suggesting that BMI as a continuous variable does not explain further variability of P-PFS scores than when BMI is used for subject categorization into obese and non-obese groups.

While these findings are supportive of a non-linear relationship between BMI and PFS, it is important to note that our clinical and non-clinical samples differed not only with regards to obesity status, but also with regards to several other characteristics, including gender, with the clinical sample comprised predominantly of female patients, as is typical in most clinical weight loss programs^24,25,53,54^. For this reason, analyses were repeated using multivariable regression models, demonstrating that, for the Food Available dimension score, the weak linear relationship with BMI, as well as group-dependent differences (clinical vs. non-clinical), were robust to adjustment by demographic variables. Adjusted logistic regression models also revealed a close to doubled odds of being in the clinical sample for each 1-point increase of this dimension score.

To address a role for unmeasured confounders in clinical vs. non-clinical comparisons, data were collected from a population-based sample, where associations between P-PFS and BMI or obesity status were mostly confirmed. Importantly, BMI-P-PFS correlations were reduced when conducted only in non-obese participants (BMI < 30.0 Kg/m^2^), and the linear associations between BMI and P-PFS scores were greatly reduced when adjusting for obesity status, considered as a binary variable. Obesity-dependent differences of P-PFS scores were also confirmed, and robust to adjustment for age, gender and education. In these multivariable logistic regression models we further confirmed that the odds of being obese approximately doubles for each 1-point increase in the P-PFS Aggregate or Food Available scores. Similar numbers were obtained when prevalence rate ratios were calculated.

To our knowledge this is the first study to fully characterize and quantify the associations between PFS and BMI, and points towards a non-linear relationship between the two variables. Our findings are consistent with prior studies addressing associations between BMI and other reward-related measures, also reporting non-linear relationships. This holds true both for behavioral (sensitivity to reward and food addiction)^39,41^ and clinical (BED)^55,56^ variables, as well as biological markers (striatal DA D2 receptor binding)^40^. With regards to food addiction symptomatology, Yale Food Addiction Scale (YFAS) scores^39^, have been shown to be low and similar across normal-weight and underweight individuals, higher in the overweight and mild obesity BMI range and reach a ceiling level in severe obesity^39^. Thus, and as is the case for hedonic hunger, positive associations between BMI and YFAS were observed only in studies comprising samples with a wide range in BMI^57^, and not found within samples of exclusively normal-weight or of severely obese individuals^39^. BED also has a clear association with obesity^58,59^, and is particularly frequent in severely obese patients seeking treatment^60^. While higher binge eating frequency has been demonstrated amongst obese women^61^, differences in BMI have not been found when comparing binge eaters with non-binge eaters among severely obese patients^39,62,63^. It is thus likely that a weak positive BMI-BED association would emerge in samples with a wide BMI range, but not in samples of exclusively obese patients^39^. Finally, some authors have suggested that the relationship between measures of reward sensitivity and BMI is consistent with a proposed inverted U-shaped relationship between dopamine tone, as inferred from DA D2R binding potential, and BMI^40^. Such a unifying framework, considering obesity status as an explanation for the relationship between reward-related measures and BMI, offers a valuable perspective on the findings reported here.

Across these three different samples, the P-PFS Food Available score was found to be most associated to the presence of obesity, while the P-PFS Food Tasted scores had little or no association. This is consistent with prior research comparing obese and non-obese individuals^25,26^, and also research in surgical^33^ and non-surgical^50^ weight-loss interventions, in which the Food Available score is the dimension that is most associated with weight loss. In fact, the construct of hedonic hunger, as assessed by the PFS, has been conceptualized to integrate two distinct components of food reward: “wanting”, and “liking”, respectively assessed by the Food Available and Food Tasted dimensions^22^. These results suggest that, in obese individuals, hedonic hunger is increased by enhanced “wanting”, which reflects appetite/incentive motivation, but not by changes of “liking”, that represents the experience of pleasure while eating^64^. Similarly, others have found evidence of an increased drive to eat, rather than enhanced pleasure during ingestion, in obese individuals^65^. Nevertheless, there is also evidence of enhanced liking for palatable foods in obesity^66^. Further research is thus necessary to clarify the relationship of PFS domain scores with the constructs of “wanting” and “liking”, ideally using non-questionnaire based behavioral measurements, such as progressive ratio tasks^67^ and food pleasantness ratings^66^. Given the importance of dopaminergic processing in the neurobiology of reward^68,69^ and the proposed role for dopaminergic dysfunction, namely decreased DA D_2_ receptor availability, in the pathophysiology of obesity^14,16^, the relationship between PFS scores and markers of dopaminergic function is also an important unresolved question.

Findings regarding the preferential association of obesity with the P-PFS Food Available dimension may also reflect a neurobiological/behavioral consequence of becoming obese, rather than a cause for obesity. While this would be consistent with proposals of an obesity-induced shift in responsiveness to food cues (conceptually integrated in Food Available and Food Present dimensions^23^) but not to food intake (conceptually integrated in Food tasted dimension^17,23^), it is nevertheless a speculative proposal, and opposing possibilities can also be considered. In fact the PFS involves the anticipatory, rather than the consummatory, phase of eating^30^. Thus, while chronic overconsumption may be more objectively reflected in body weight, exacerbated anticipatory factors could also occur in individuals within the normal-weight range, and contribute to future weight gain. This could dilute the association between hedonic hunger and obesity, and sustain findings of potentially larger differences between obese and non-obese individuals for other eating-related consummatory measures, namely of disinhibition and emotional eating^70^. A better clarification for the position of the PFS as reflection of current obesity status vs. a risk factor for obesity will thus require further longitudinal research. In any such future research projects, the results reported here should be carefully considered since use of BMI, rather than obesity status, could confound findings.

While the findings reported here provide important contributions to disambiguate the associations of hedonic hunger with BMI and obesity, they should be interpreted in the context of the study design. Importantly, and as mentioned above, the non-clinical and clinical samples can be presumed to have sampling bias, such as gender bias. Nevertheless, the findings obtained in the clinical and non-clinical samples were robust to adjustment for demographic variables, namely gender, age and education, which did not seem to confound the relationship of P-PFS scores to obesity status and BMI. Furthermore, findings in the clinical vs. non-clinical comparison were confirmed in a population-based sample, in which a gender sampling bias is of less concern, and also found to be robust to adjustment for demographic variables. Nevertheless, obese and non-obese samples are likely to differ in several other characteristics, such as BED prevalence, LOC eating and severe emotional distress^71^. Hedonic hunger is associated with both development and maintenance of LOC eating^9,30^, raising the possibility that the group differences described are determined by differences in LOC eating, among other unmeasured biological or behavioral characteristics.

It is also important to consider that, in the non-clinical and in the population samples, weight and height was self-reported, which could be a source of measurement error. Indeed, the prevalence of obesity in Portugal^72^, is slightly above that estimated in our population sample (14.2% vs. 12.8%), which is consistent with evidence that weight is underestimated when self-reported^73^. However there is also evidence for a strong correlation between objectively measured and self-reported anthropometric data^74^. Furthermore, our results rendered similar measurements of the relationship between P-PFS scores and BMI and obesity status across distinct populations. Finally, data collection was performed in a cross-sectional sample, allowing for measurements of association, but not causality, in the relationship between BMI and P-PFS. It is vital that, in future research, longitudinal assessments are performed, to address this critical question.

In conclusion, our study has revealed that hedonic hunger is a relevant moderating factor for obesity status. Although hedonic hunger levels, as measured by the P-PFS score, were only weakly associated with BMI, the Food Available dimension score was robustly associated with the presence of obesity. In fact, we found that the odds of being obese increase approximately 2 times for each unit increase in the P-PFS Food Available score, even when adjusting for several sociodemographic variables. Our findings should inform the use of the PFS, and other reward-related measures, in studies with specific samples, such as obese patients, in which associations between BMI and such measures are usually assessed using correlation analyses. This study also provides insight on the potential application of the PFS as obesity marker in future studies aiming to explore hedonic hunger in the context of obesity and its treatment. Importantly, this study reinforces the need to phenotype obesity beyond BMI, which could be partially achieved through the use of psychometric measures such as the PFS.

## Electronic supplementary material

Supplementary Information
