# Supplementary material for: Association between hedonic hunger and body-mass index versus obesity status
Source: Sci Rep. 2018 Apr 11;8:5857. doi: 10.1038/s41598-018-23988-x (PMC5895788; doi:10.1038/s41598-018-23988-x)
Supplement: Supplementary file 1 — Supplementary Information [file 41598_2018_23988_MOESM1_ESM.pdf]

Suplemmentary Information

Association between hedonic hunger and body-mass index versus obesity status.

Gabriela Ribeiro[a, b, #], Marta Camacho[a, #], Osvaldo Santos[c, d], Cristina Pontes[e], Sandra Torres[f], Albino J. Oliveira-Maia[a, g, h, i, *]

[a] Champalimaud Clinical Centre,

Champalimaud Centre for the Unknown,

Av. de Brasília, Doca de Pedrouços,

1400-038 Lisboa, Portugal.

[b] Lisbon Academic Medical Centre PhD Program,

Faculdade de Medicina, Universidade de Lisboa,

Avenida Professor Egas Moniz,

1649-028 Lisboa, Portugal.

[c] Instituto de Saúde Ambiental,

Faculdade de Medicina, Universidade de Lisboa,

Avenida Professor Egas Moniz,

1649-028 Lisboa, Portugal.

[d] Instituto de Medicina Preventiva e Saúde Pública,

Faculdade de Medicina, Universidade de Lisboa,

Avenida Professor Egas Moniz,

1649-028 Lisboa, Portugal.

[e] Psychiatry and Mental Health Clinic,

Centro Hospitalar de São João,

Alameda Prof. Hernâni Monteiro,

4200-319 Porto, Portugal.

[f] Faculdade de Psicologia e de Ciências da Educação,

Universidade do Porto,

Rua Alfredo Allen,

4200-135 Porto, Portugal.

[g] Department of Psychiatry and Mental Health,

Centro Hospitalar de Lisboa Ocidental,

Rua da Junqueira, 126,

1340-019 Lisboa, Portugal.

[h] NOVA Medical School | Faculdade de Ciências Médicas,

Universidade Nova de Lisboa,

Campo Mártires da Pátria 130,

1169-056 Lisboa, Portugal

[i] Champalimaud Research,

Champalimaud Centre for the Unknown,

Av. de Brasília, Doca de Pedrouços,

1400-038 Lisboa, Portugal.

# these authors contributed equally to this work

Supplementary Table 1. Associations between BMI and P-PFS scores, tested in the clinical and non-clinical samples using sequential multivariable linear regression models.

Variable	**Model 1** (R^2=0.57)		**Model 2** (R^2=0.57)		**Model 3** (R^2=0.57)		**Model 4** (R^2=0.56)		**Model 5** (R^2=0.57)	
	β	p	β	p	β	p	β	p	β	p
**Gender**	0.45	0.6	0.48	0.6	0.52	0.5	0.47	0.6	0.46	0.6
**Age**	1.51	<0.001	1.52	<0.001	1.52	<0.001	1.52	<0.001	1.5	<0.001
**Age^2**	-0.01	<0.001	-0.01	<0.001	-0.01	<0.001	-0.01	<0.001	-0.01	<0.001
**Education**	-2.13	0.001	-2.1	0.001	-2.1	0.001	-2.1	0.001	-2.1	0.001
**Education^2**	0.08	0.003	0.08	0.003	0.08	0.003	0.08	0.003	0.08	0.003
**P-PFS** - Aggregate			0.56	0.2						
**P-PFS** - Food Available					1.02	0.01				
**P-PFS** - Food Present							0.17	0.6		
**P-PFS** - Food Tasted									-0.39	0.3

Supplementary Table 2. Associations between group status (clinical vs. non-clinical) and P-PFS scores, tested in the clinical and non-clinical samples using sequential multivariable logistic regression models.

Variable	**Model 11** (c=0.96)			**Model 12** (c=0.96)			**Model 13** (c=0.96)			**Model 14** (c=0.96)			**Model 15** (c=0.96)		
	β	p	OR (95% CI)	β	p	OR (95% CI)	β	p	OR (95% CI)	β	p	OR (95% CI)	β	p	OR (95% CI)
**Gender**	-1.44	0.003	0.2 (0.09-0.6)	-1.45	0.002	0.2 (0.09-0.6)	-1.47	0.002	0.2 (0.09-0.6)	-1.42	0.002	0.2 (0.097-0.6)	-1.43	0.002	0.2 (0.096-0.6)
**Age**	0.5	<0.01	1.7 (1.3-2.1)	0.51	<0.01	1.7 (1.3-2.2)	0.51	<0.01	1.7 (1.3-2.2)	0.51	<0.01	1.7 (1.3-2.2)	0.5	<0.01	1.6 (1.3-2.1)
**Age^2**	-0.004	0.02	0.996 (0.993-0.999)	-0.004	0.02	0.996 (0.992-0.999)	-0.004	0.03	0.996 (0.992-1)	-0.004	0.02	0.996 (0.992-0.999)	-0.004	0.02	0.996 (0.993-0.999)
**Education**	-0.57	0.049	0.6 (0.3-0.99)	-0.54	0.06	0.6 (0.3-1.02)	-0.51	0.08	0.6 (0.3-1.06)	-0.55	0.05	0.6 (0.3-1.01)	-0.57	0.05	0.6 (0.3-1.002)
**Education^2**	0.03	0.03	1.03 (1.003-1.06)	0.03	0.04	1.03 (1.002-1.06)	0.03	0.05	1.03 (1-1.05)	0.03	0.03	1.03 (1.002-1.06)	0.03	0.03	1.03 (1.003-1.06)
**P-PFS -** Aggregate				0.4	0.08	1.5 (0.95-2.5)									
**P-PFS -** Food Available							0.6	0.008	1.8 (1.2-2.8)						
**P-PFS -** Food Present										0.22	0.2	1.3 (0.9-1.8)			
**P-PFS -** Food Tasted													-0.1	0.6	0.9 (0.6-1.4)
